# Pyogenic granuloma in relation to dental implants: Clinical and histopathological findings

**DOI:** 10.4317/jced.52094

**Published:** 2015-10-01

**Authors:** Eduardo Anitua, Laura Pinas

**Affiliations:** 1 Private practice in implantology and oral rehabilitation in Vitoria, Spain; 2 Eduardo Anitua Foundation, Vitoria, Spain

## Abstract

**Background:**

The occurrence of pyogenic granuloma in association to dental implants is rare and only five cases have been reported in the literature.

**Material and Methods:**

Patients charts were analyzed to select patients who had been diagnosed for pyogenic granuloma and its association with dental implants had been evaluated. The clinical status of the dental implants and the prosthesis had also been assessed.

**Results:**

Clinical and histopathological diagnosis of pyogenic granuloma had been reached for soft mass growth in association with dental implants in 10 patients. Histological analysis of all samples was performed to obtain a firm diagnosis of finding against pyogenic granuloma lesions. Accumulation of dental plaque due to poor oral hygiene and improper design of the prosthesis had been related to the occurrence of pyogenic granuoloma. This lesion showed no predilection to specific surface type and had no significant association with marginal bone loss.

**Conclusions:**

Pyogenic granuloma should be included in the differential diagnosis of soft mass growth around dental implants.

** Key words:**Reactive lesion, soft mass, pyogenic granuloma, dental implant, titanium.

## Introduction

Gingival reactive lesions like pyogenic granuloma have frequent occurrence around natural dentition, however, their association with dental implants is not common. The causes of pyogenic granuloma (PG) in relation to dental implants are not clear mainly due to few published cases (1-8).

Tooth-related PG is a result of tissue response to minor injury or chronic low-grade irritation (9-16). Clinically, oral PG is characterized as a soft mass of smooth or lobulated appearance that could be sessile or pedunculated and frequently presents ulceration. The lesion grows rapidly for a few weeks and the colour ranges from pink to red purple and haemorrhage may occur either spontaneously or after minor trauma (8). Its incidence is relatively common and accounts for 3.81-7% of all biopsies harvested from the oral cavity (13-16).

Microscopically, the lesion is characterized by prominent capillary growth in hyperplastic granulation tissue, which suggests a strong activity of angiogenesis. The blood vessels often show a clustered or medullary pattern separated by less vascular fibrotic septa, leading some authorities to consider PG as a polypoid form of capillary hemangioma (17).

The lesions of PG may be found in the oral cavity or extraorally. The most frequent intraoral localization is the gingiva (about 60-70%), but lesions can occur on the lips (14%), tongue (9%), buccal mucosa (7%) and palate (2%) (18-24). Possible treatment methods are excision, curettage, cryotherapy, sclerotherapy, chemical and electrical cauterization, cryotherapy and the use of lasers with the carbon dioxide (CO2) or argon (25-29). Conservative local excision is the preferred form of treatment and recurrence rates after excision range from 0% to 16% (29).

However, to the best of the authors’ knowledge, only 5 cases of pyogenic granuloma in association with a dental implant have been reported in the international literature (1-3,7,8). Within the context of the scarce information available on these lesions, the aim of the present study was to report 10 novel clinical cases of pyogenic granuloma in association with titanium dental implants and to elucidate potential risk factors. Finally, the presence of marginal bone loss was evaluated.

## Material and Methods

Patients charts at the service of oral medicine of Anitua’s Dental Clinic (Alava, Spain) were revised from 1991 to 2011. Patients selection was based on the following inclusion criteria:

• Treatment of pyogenic granuloma.

• The presence of histopathological diagnosis.

• Lesion in relation to dental implants.

All patients who did not fulfill all inclusion criteria were excluded from the study.

Data were collected to report on patient age, gender, patient´s disease, lesion site, type of dental implant (surface and morphology), predisposing factors (trauma, prosthesis type, poor oral hygiene), clinical and radiographic features, diagnosis, treatment and recurrence. Orthopantomography (OPG) of all lesions were examined to compare the presence or absence bone resorption around dental implants.

A descriptive statistical analysis of all variables were performed. Then the relationship between PG and marginal bone loss was analyzed by nonparametric Spearman correlation. The effect of surface type on marginal bone loss was also analyzed with one-way ANOVA and Levene post hoc test. The statistical significance was set at *p*-value < 0.05. All the statistical analyses were performed using the SPSS v15.0 for Windows statistical software package (SPSS Inc., Chicago, IL, USA).

## Results

Ten patients with pyogenic granuloma in relation to dental implants had been identified. They were 2 males and 8 females. Patients’ age ranged from 21 to 92 years and all were non-smokers. Five of the ten patients (50%) had systemic disorders: cardiac arrhythmia (1 patient), hypertension (2 patients), atrial fibrillation (2 patients), Type II diabetes mellitus (2 patients), hepatitis C (1 patient ), hypothyroidism (1 patient). Within the group of patients with systemic disease, 3 of them were using 1 to 2 drugs daily, whereas the remaining patient took more than 2 drugs.

With regard to oral hygiene habits, 20% of patients reported to brush once a day, 50% did twice daily and 30% brushed three times a day. A 90% of the patients received professional prophylaxis twice a year and the other 10% once a year. In the use of hygiene products the obtained results were as follows: a) use of mouthwash: only was used by 3 patients (37.5%), b) use of dental floss: only one patient (12.5%), and c) interproximal brushes: 3 patients (37.5%).

The distribution of PG lesions was even between maxilla and mandible (50% for each region), and the most common oral site affected by PG was the area of tooth 41 (2 cases).

The development of PG was related to only accumulation of dental plaque (one patient), bad prosthetic design (one patient), and both factors (one patient). In 4 patients, there had been a combination of tissue pressure by the prosthesis and poor oral hygiene. However, no etiological factor could be related to the development of PG in 3 patients. The clinical size of the lesions ranged from 1.1 x 0.6 mm to 36 x 19 mm. The mean diameter was 7.2 mm. All the lesions were excised and sent for histological examination. The defects were covered with a autologous fibrin membrane (Anitua’s protocol). During the first week after the operation, all patients were given analgesic and 0.2% chlorhexidine gluconate mouthwash. During the follow-up period (range two months to 10 years), there were no recurrences.

**Figure 1:**
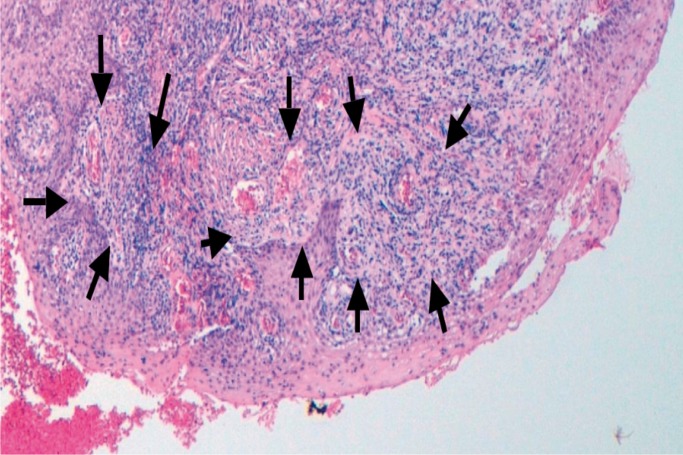
Histological images of the pyogenic granuloma showing an appearance similar to granulation tissue. The histological type of the pyogenic granuloma is non-lobular capillary hemangioma. Arrow heads label blood vessels surrounded by connective tissue.

The histopathological reports indicated the diagnosis of PG and the description of highly vascular proliferation that resembles granulation tissue (Fig. 1).

**Figure 2:**
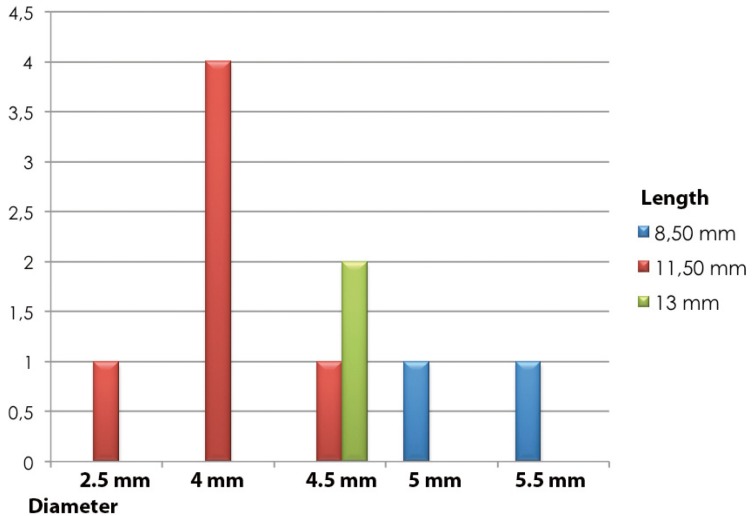
Diameter and length of dental implants related to the pyogenic granuloma.

The surfaces of the implants associated with the lesion were smooth (2 implants), machined (3 implants) and rough (5 implants). In no case there was a natural tooth adjacent to the implants related to the lesion. The characteristics of diameters and lengths of the implants studied can be seen in figure 2. The average load time of the implants studied was 115 months (SD = 67.5), ranging from a range of 9 to 184 months. Oral rehabilitation was performed with complete prosthesis in 9 patients. The mean mesial bone loss was 2.14 mm (range 0 to 6.50 mm, SD = 2.07) and the mean of distal bone was 1.66 mm (range 0 to 3.75 mm, SD = 1.21.

**Figure 3:**
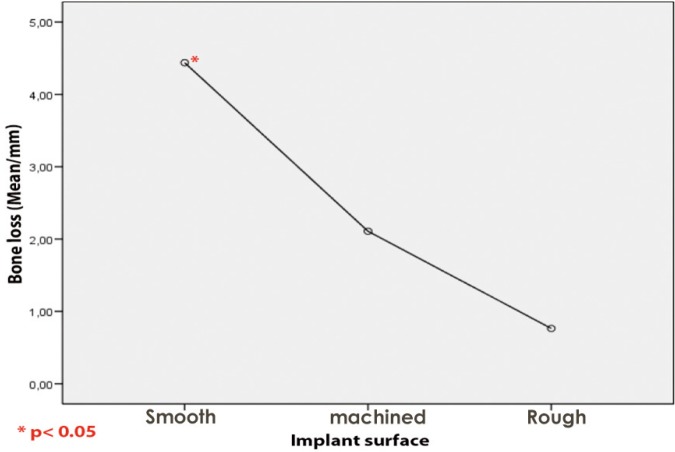
Peri-implant bone loss grouped by type of surface. The bone loss was the highest for implants with smooth surface.

There were no statistically significant association between the PG area and the marginal bone loss. However the smooth implant surface showed a significant influence on bone loss (Anova: *p* = 0.001) (Fig. 3).

## Discussion

The clinical and histopathological findings have confirmed the diagnosis of pyogenic granuloma in 10 patients. The present study is the one with the highest number of implant-related PG lesion that are available until now in the scientific literature. These PG lesions have been diagnosed as non-lobular capillary hemangioma. There are two histological types of PG. The first type is characterized by proliferating blood vessels that are organized in lobular aggregates. This histological type of PG was called lobular capillary hemangioma (LCH type). The second type (non-LCH type) consist of highly vascular proliferation that resembles granulation tissue (1,4,11).

Literature data indicated that PG is rarely associated with dental implants, as there are only five cases reported (1-3,7,8). However, other reactive lesions such as gingival hyperplasia caused by phenytoin, allergy to titanium abutments or peripheral giant cell granulomas have been reported in the international literature. Causes of conventional oral pyogenic granulomas are not clear, although it has been shown that different stimuli irritants that can trigger them, such as repeated trauma, poor oral hygiene and hormonal problems (1-20). About 30-50% of patients with PG have a history of local trauma (9).

Considering PG, in the case reported by Dojcinovic *et al.* (1), the inappropriate healing cap has resulted in dental plaque accumulation and chronic inflammation of the peri-implant tissues, triggering the development of a PG. However this was not the cause for PG in the case reported by Olmedo *et al.* (2). The authors have pointed out to the presence of “metal-like” particles and have postulated that these particles could be the result electrochemical phenomena, corrosion, friction, or a synergistic combination of these events (4,5). Once released, these particles may trigger an inflammatory response mediated by cytokines and macrophages (5). This inflammatory reaction could perpetuate the pseudo-periodontal pocket that generates the lesion around the implant (5).

In the case reported by Etöz *et al.* (3), the presence of a gap between the alveolar bone and implant surface could be associated with the occurrence of pyogenic granuloma. Although bone splitting technique was adequately performed, trauma from the upper dentition and lack of adequate keratinized mucosa could result in soft tissue invasion and may have been responsible for PG development (3). Kang *et al.* (8) have stated that the causes of the occurrence of PG was unclear however, the antithrombotic therapy may have some involvement in the development of the lesion.

In this study, PG was related to only accumulation of dental plaque (one patient), bad prosthetic design (one patient), and to both factors (one patient). The mean age of patients was 74.5 years and most of them have decreased manual dexterity. The bad prosthetic design with flanges could difficult the maintenance of good oral hygiene and could predispose the development of PG around dental implants. In 4 cases, there has been a combination of tissue pressure by the prosthesis and poor oral hygiene. However, no etiological factor could be related to the development of PG in 3 patients where implants have a smooth surface. Previously published studies have reported the association of PG to implant with roughened surface (1-3,7,8). There are no published data on smooth or machined surfaces.

In the histological analysis, the presence of metal-like particles were searched for. Such particles could not be found in any of the 10 biopsies and thus could not be related to the development or progression of PG.

The marginal bone loss has shown no association with the presence of PG. However, Implant’s surface has affected significantly the marginal bone loss around dental implants. This results can indicate that PG showed no predilection to specific surface type.

With the data obtained from this study and others in the literature we can conclude that pyogenic granuloma in association with dental implants seems to respond to the same stimuli that triggers tooth-related PG. This lesion should be included in the differential diagnosis of soft mass growth around dental implants. PG had no significant correlation with the marginal bone loss around dental implants.
